# CDHR3 extracellular domains EC1-3 mediate rhinovirus C interaction with cells and as recombinant derivatives, are inhibitory to virus infection

**DOI:** 10.1371/journal.ppat.1007477

**Published:** 2018-12-10

**Authors:** Kelly Watters, Ann C. Palmenberg

**Affiliations:** Institute for Molecular Virology, Department of Biochemistry, University of Wisconsin-Madison, Madison, Wisconsin, United States of America; University of Maryland, UNITED STATES

## Abstract

Viruses in the rhinovirus C species (RV-C) are more likely to cause severe wheezing illnesses and asthma exacerbations in children than related isolates of the RV-A or RV-B. The RV-C capsid is structurally distinct from other rhinoviruses and does not bind ICAM-1 or LDL receptors. The RV-C receptor is instead, human cadherin-related family member 3 (CDHR3), a protein unique to the airway epithelium. A single nucleotide polymorphism (rs6967330, encoding C529Y) in CDHR3 regulates the display density of CDHR3 on cell surfaces and is among the strongest known genetic correlates for childhood virus-induced asthma susceptibility. CDHR3 immunoprecipitations from transfected or transduced cell lysates were used to characterize the RV-C interaction requirements. The C529 and Y529 variations in extracellular repeat domain 5 (EC5), bound equivalently to virus. Glycosylase treatment followed by mass spectrometry mapped 3 extracellular N-linked modification sites, and further detected surface-dependent, α2–6 sialyation unique to the Y529 format. None of these modifications were required for RV-C recognition, but removal or even dilution of structurally stabilizing calcium ions from the EC junctions irreversibly abrogated virus binding. CDHR3 deletions expressed in HeLa cells or as bacterial recombinant proteins, mapped the amino-terminal EC1 unit as the required virus contact. Derivatives containing the EC1 domain, could not only recapitulate virus:receptor interactions *in vitro*, but also directly inhibit RV-C infection of susceptible cells for several virus genotypes (C02, C15, C41, and C45). We propose that all RV-C use the same EC1 landing pad, interacting with putative EC3-mediated multimerization formats of CDHR3.

## Author summary

The RV-C species was first identified in 2006 and is a major cause of severe respiratory illnesses in children and hospitalization for asthma exacerbations. Refractive to growth in standard tissue culture, RV-C uses a receptor that is distinct from the RV-A or RV-B species, which we identified as CDHR3 in 2015. Here, we developed and used *in vitro* pull-down assays to the determined biochemical and structural features of CDHR3 that are important for virus binding. Additionally, we purified soluble recombinant CDHR3 proteins that could directly bind virus and inhibit RV-C infection. The assays and biological materials reported here are promising and useful tools for RV-C-specific antiviral drug development.

## Introduction

Rhinoviruses (RV) are a large group of non-enveloped, single-stranded, positive-sense RNA viruses in the *Enterovirus* genus of the *Picornaviridae* family. There are more than 160 genotypes, more or less synonymous with serotypes, classified into three species, the RV-A, RV-B, and RV-C. Collectively, RV are the primary etiological agents of upper respiratory tract common colds, but many also induce more severe lower respiratory tract illnesses, including bronchitis and pneumonia [1–3]. The RV-A and RV-C isolates are known to cause more severe wheezing illnesses compared to those of the RV-B, but the RV-C are of additional clinical interest because of their close association with acute asthma exacerbations in children [4–8].

The RV-C were first described in 2006 [9], but not grown in culture until 2011 [10]. Initially, virus propagation was restricted to sinus mucosa organ tissues or to airway epithelial air-liquid-interface cultures (ALI) because the isolates proved refractive to standard cell culture [11,12]. These labor-intensive, low virus-titer systems did allow the determination that RV-C growth required a cellular receptor component distinct from intercellular adhesion molecule 1 (ICAM-1) or the low-density lipoprotein receptor (LDLR) used by the RV-A/B species [10,13,14]. Gene expression profiles from susceptible and non-susceptible cells subsequently identified cadherin-related family member 3 (CDHR3) as the missing cell-surface constituent [15].

CDHR3 belongs to the cadherin superfamily of transmembrane calcium-dependent adhesion proteins. The better-described classical cadherins are expressed in a variety of tissues, where they mediate cell-cell interactions, usually through homologous protein contacts, or where they participate in cell signaling, epithelial polarity, and tissue development and organization [16–19]. CDHR3 expression, in contrast, is generally restricted to airway tissues, with protein display primarily on the apical surfaces of ciliated epithelial cells [20,21]. The biological role of CDHR3 in lung development or function is unknown. Although the gene locus generically shows a high degree of sequence conservation among all animal genomes, comparative human genetics records a unique non-synonymous single nucleotide polymorphism (SNP) RS6967330 [22], converting a strongly conserved ancestral residue, Tyr529 (TAT, Y529) to cysteine (TGT, C529). Transfected cells, or primary cultures derived from homozygous or heterozygous human allele carriers differentially display these sequences, with the Y529 protein observed at a higher surface density than C529, for the same amount of gene expression [23]. The Y529-encoding allele, even though it is ancestral [24], is the minor frequency sequence in modern humans. The 3–15% of carriers, especially children, have significantly stronger genetic associations with severe asthma exacerbations, and in particular, those trigged by RV-C infections [22,25].

**Fig 1:**
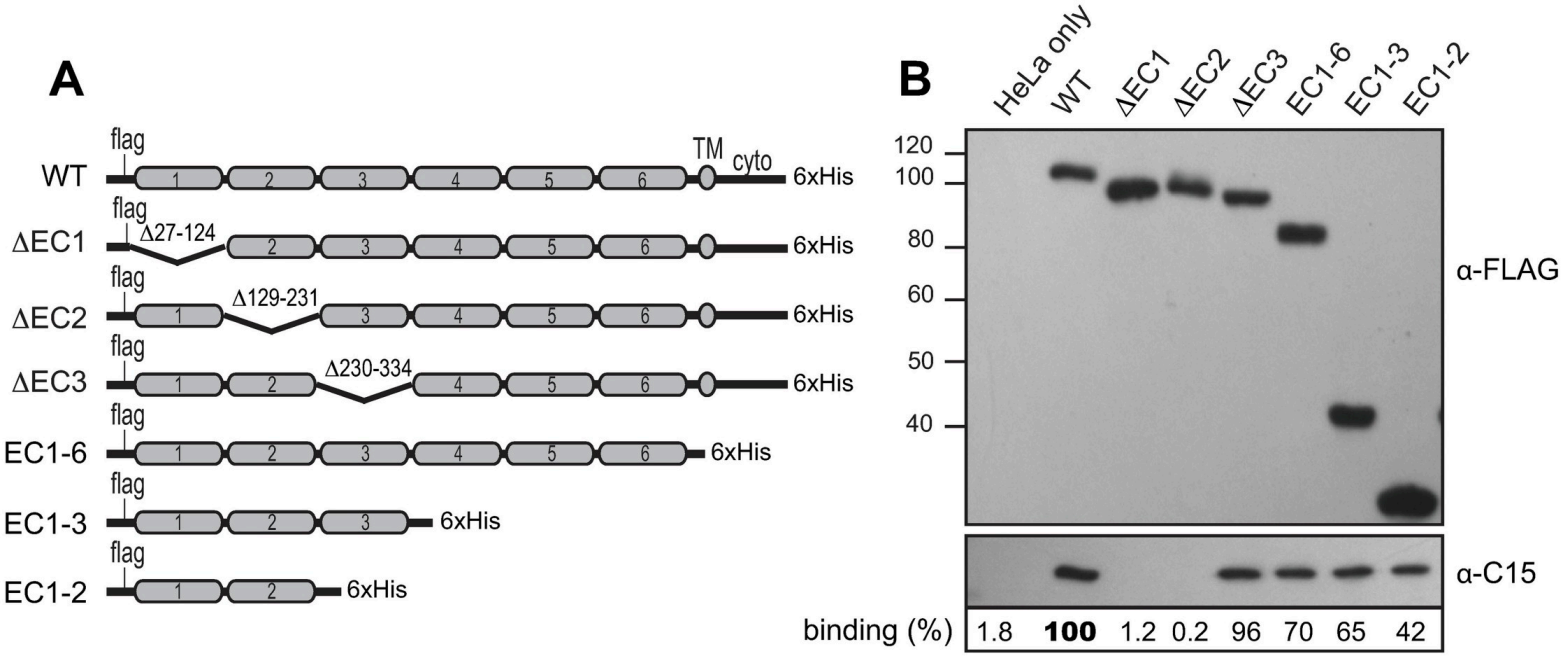
Mapping RV-C binding domains in CDHR3. (A) Design of CDHR3 EC deletion constructs. (B) Cell lysates of HeLa cells transfected with these cDNAs were reacted with sucrose purified C15 virus (10^7^ PFUe) as described in Methods. Western assays after immunoprecipitation with an α-His mAb detected CDHR3 (via α-FLAG) and captured virus (α-C15). Binding % is the observed C15 signal pixel count normalized to the CDHR3 protein (α-FLAG) signal in the panel above (Total Lab 100) relative to the WT CDHR3 lane (bold face).

A defining feature of all cadherins is their distinctive rod-like arrangement of linear tandem-repeat extracellular domains (EC). Collectively or individually, these units mediate the various *cis* and *trans* contacts needed for adhesion specificity [26]. Each domain of about 110 amino acids is distinct in sequence, but they typically configure into similar 7-stranded, antiparallel “Greek key” motifs. The linked repeat units (usually 5) are preceded by a signal sequence and followed with a short transmembrane segment and a cytoplasmic tail. Calcium ions (2 or 3) chelated by multiple acidic residues, are set into each EC junction, and required for relative domain orientation as well as the overall rigidity of the long, slightly curved, rod-like conformations. Homotypic and heterotypic cadherins interact in *cis* (parallel orientation, same cell) and/or *trans* (anti-parallel orientation, opposing cell) by reciprocal ionic contacts on their various EC surfaces to provide adhesion functions [27]. The CDHR3 sequence (885 amino acids) encodes six EC repeats with the usual, easily defined amino and carboxyl extensions (Fig 1). The Y529/C529 dichotomy is in EC5, predicted structurally at the interface with EC6, possibly affecting the calcium stability of that junction [23]. A correlate protein docking model relying on the recent structure resolution of RV-C15a [28] suggested that CDHR3 may interact with this virus exclusively through contacts in the first two domains, assuming a binding orientation that could putatively accommodate receptor monomers or *trans*-dimers [15].

These predictions, though, are strictly computational. Validation of any model, in lieu of an authentic co-structure, required formal characterization of actual RV-C interactions with multiple native and recombinant formats of CDHR3. We report here the development of relevant biochemical pull-down assays, leading to determination of the minimum forms of CHDH3 capable of direct virus interactions. As soluble recombinant materials, the required protein units, rEC1, rEC1-2, rEC1+3(Δ2) or rEC1-3, when properly folded in the presence of Ca^++^, could recapitulate virus:receptor binding interactions and could inhibit RV-C infection of susceptible cells for at least 4 different virus genotypes.

## Results

### Virus binding to cell-expressed CDHR3

**Fig 2:**
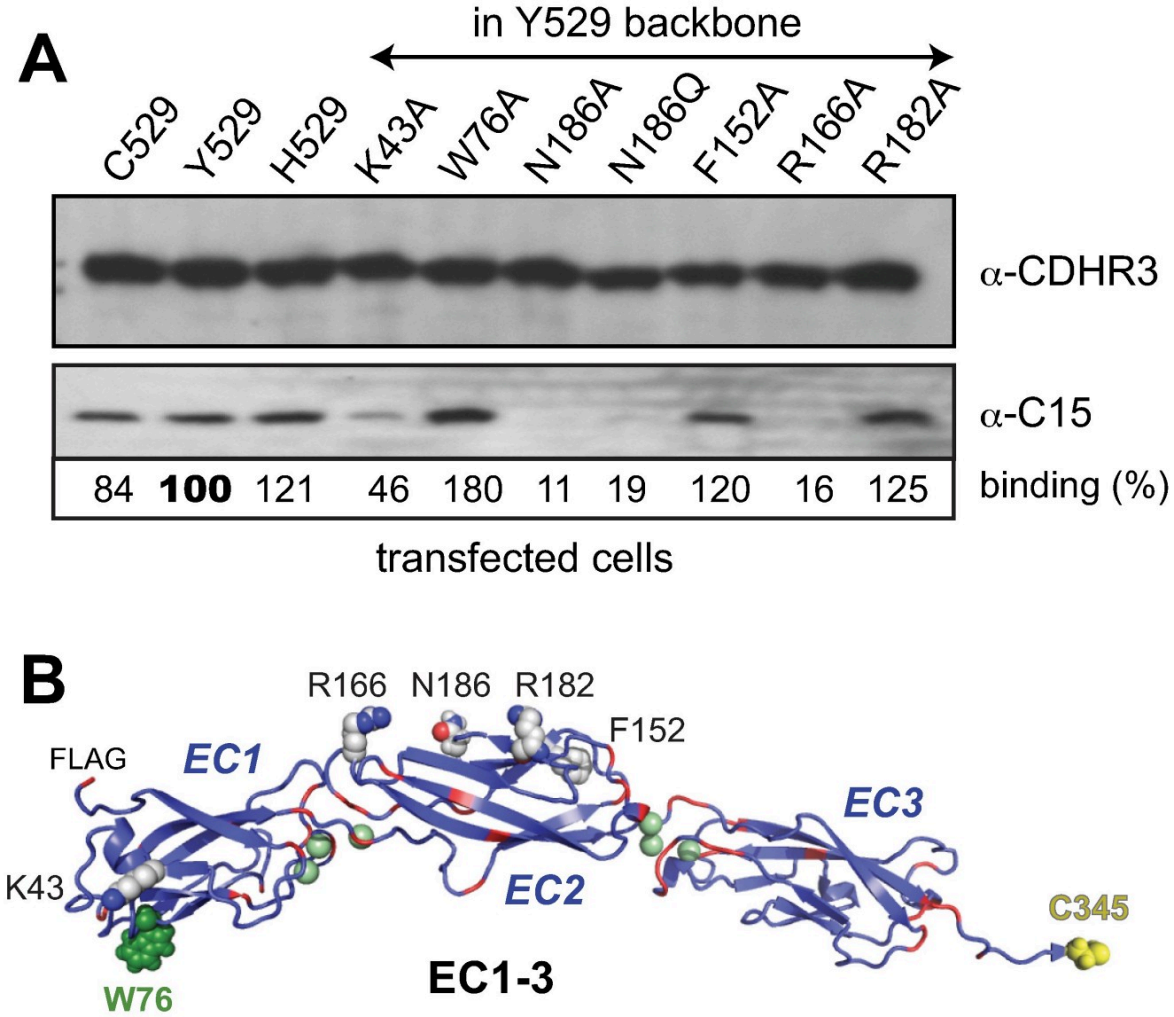
CDHR3 mutations. (A) Lysates from HeLa cells transfected with full-length CDHR3 cDNAs encoding the specified point mutations were reacted with C15 virus. Immunoprecipitation and protein detection used an α-CDHR3 mAb reactive with the cytoplasmic domain. Captured virus was quantitated as in Fig 1. (B) PyMol depiction of computed model by Robetta [23,56] of rEC1-3 segments show putative locations of mutations. Green spheres predict calcium binding sites, red depicts Asp+Glu sequences.

RV-C will bind and infect HeLa cells that are transfected or transduced for surface expression of full-length CDHR3 sequences [15]. During transfections however, the exterior protein presentation and therefore formal virus access is dependent on each individual cell’s cDNA uptake as well as that sequence’s innate display potential (e.g. C529 vs Y529). Transduced cells introduce additional surface variabilities by the very nature of clonal selection. A reproducible assay for virus binding, dependent only on the introduced CDHR3 sequence, was achieved by reacting C15 virus with whole-cell lysates, after transfection or transduction of preferred cDNAs. Except for the CDHR3 glycosylation status (see below) there was no indication in any experiment of virus preference for intra- or extracellular protein pool locales. Indeed, even the predominantly non-surface C529 materials readily interacted with virus, if given the chance as lysate extracts (e.g. see Fig 2a).

The initial application of this assay tested the overall EC domain requirements (Fig 1). Immunoprecipitations (IP) with mAbs to the CDHR3 carboxyl His tag (α-His) readily extracted virus if the transfections were with full-length, wild-type (WT) sequences. In agreement with the predicted interaction model, engineered removal of the entire gene segment downstream of EC1-2, including EC3-6, the TM and cytoplasmic domains, produced protein fragments (EC1-2), which remarkably, were still capable of reacting with virus. Consistent with this, internal deletions targeting the precise, short inter-domain linkers that removed just EC1 (i.e.ΔEC1), or just EC2 (i.e. ΔEC2), but not EC3 (i.e. ΔEC3), created proteins that failed to extract virus. When the IP antibody was switched to α-FLAG, reactive with the amino-proximal sequences, no tested protein co-extracted virus, although all were recognized by the mAb (Fig 1b). Therefore, while the first two extracellular domains (EC1-2) alone were sufficient to facilitate C15 binding, the process could be interfered with if the chosen IP mAb was sterically close to EC1, or in this case, the 8 amino acids adjacent upstream. A similar distance downstream of EC2 was apparently not inhibitory if bound by the α-His mAb.

Looking more closely at this region, the RV-C binding model suggested that within the EC1-2 sequence, Asn186 (N186) was a likely candidate for a putative sugar ligand contribution. Initially, this was considered of special importance because the C15a virion structure differs from RV-A/B, in that it has an unusual, species-conserved surface pocket, seemingly analogous to the sialic acid interaction site used by enterovirus D68 to mediate that receptor recognition [28,29]. Accordingly, N186 and 5 other amino acids modeled as putative EC1-2 surface features were tested in the lysate-binding assay (Fig 2). The Asn site, when replaced with Ala or Gln, gave proteins that reacted poorly with virus (reduced to 11% and 19% respectively). The same was true for EC1 domain K43A and EC2 domain R166A changes (to Ala), where binding was also markedly diminished (to 46% and 16%). Other changes though, like F152A and R182A (EC2), and particularly W76A (EC1), if anything, promoted virus IP. The W76A enhancement in particular, was repeatable in multiple experiments and CDHR3 contexts, usually averaging ~50–80% more virus pull-down than comparable WT sequences. Classical cadherins multimerize by intercalation of an amino-proximal conserved Trp into a hydrophobic pocket of the homotypic or heterotypic partner [30–32]. The CDHR3 W76, while not amino-terminal, is the only tryptophan within EC1-4. The precise quaternary arrangement of full-length CDHR3 in cells (or lysates) is difficult to assay at present. If CDHR3 W76 also participated in multimeric contacts, IP enhancement could well indicate that RV-C prefers lower order monomer or dimer receptor formats. This point was probed further in the design and subsequent testing of bacterial produced recombinant proteins.

### CDHR3 glycosylation

**Fig 3:**
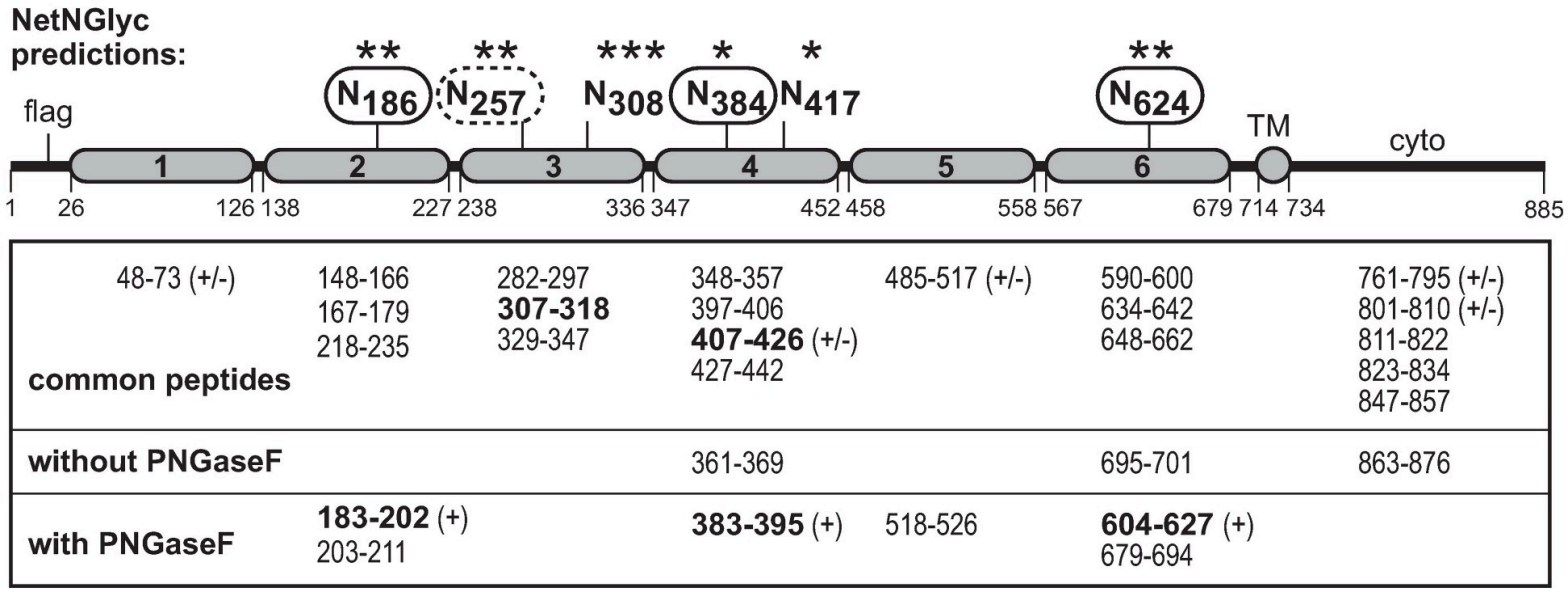
CDHR3 glycosylation. HeLa cell lysates from cells transfected with cDNA for full-length CDHR3 (Y529) were gel fractionated. The CDHR3 band was treated (or not) with PNGaseF before trypsin digestion and analysis by mass spectrometry. Identified peptides are indicated: (+) deamide form of peptide; (+/-) amide and deamide forms. NetNGlyc strength is according to primary sequence data (* is low, *** is high). Protein numbering is from GenBank AIC58018, with EC delineation from the current structure model [23].

The N186 mutation data fit the binding model’s putative expectations that glycosylation at this site might contribute to virus interactions. Almost all classical cadherins are post-translationally modified with glycans [31,33–35]. The sequence of CDHR3 predicts 6 possible N-linked sites (Fig 3). O-linked sites cannot be projected with reliability. FLAG-CDHR3 Y529 protein from transfected HeLa cells was gel-fractionated before and after treatment with PNGaseF. Mass spectrometry then identified the trypsin-dependent peptide differences. PNGaseF reactions deaminate culpable Asn to Asp in the process of removing glycans so the peptide charge relative to sequence and new peak appearance are diagnostic of prior glycosylation [36]. The recovered CDHR3 peptides analyzed this way recapitulated ~80% of the sequence, definitively identifying N186, N384, and N624 as N-linked glycosylation sites. Fragments for N308 and N417 did not respond to enzyme treatment, nor was their amination status changed, indicating that at least for transfection-derived materials, these sites are not glycosylated. The modification status of N257 could not be determined because the appropriate fragment (40 amino acids) was not recovered from either treatment condition.

**Fig 4:**
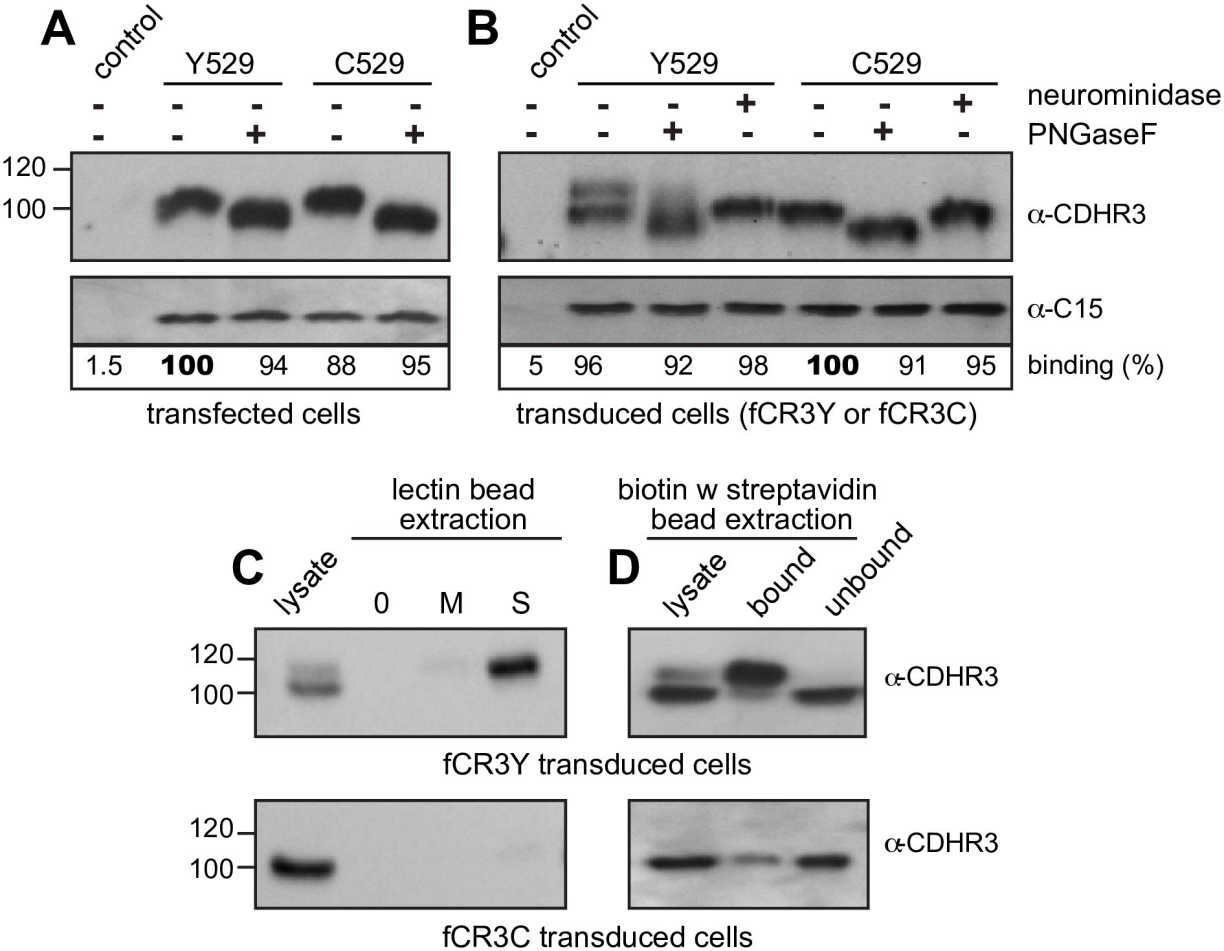
Virus binding and CDHR3 glycosylation. Lysates from HeLa cells transfected (A) or transduced (B) with full-length cDNAs encoding Y529 or C529 CDHR3 proteins were reacted with C15 virus in the presence or absence of PNGaseF or neuraminidase as described in Methods. Immunoprecipitation and protein detection used an α-CDHR3 mAb reactive with the cytoplasmic domain. Captured virus was quantitated as in Fig 1. (C) As in B, transduced cell lysates were treated (or not) with biotinylated *Sambucus nigra* (S) or *Maackia amurensis* (M) lectins before extraction with streptavidin agarose beads and protein detection with α-CDHR3. (D) Plated stably transduced HeLa cells expressing FLAG-Y529 or C529 CDHR3 were biotinylated and labeled cell surface proteins were isolated with streptavidin agarose beads.

CDHR3 human protein variants Y529 and C529 from transfected HeLa cells migrate identically during gel fractionation (Fig 2a), as does His529 (H529), a naturally occurring positional mutation found in a few other mammalian genomes like mice and horses [23,24]. Despite a differential surface exposure on intact cells [15] the co-migration of transfected Y529 and C529 was mirrored by equivalent band shifts after PNGaseF treatment of ~8 kDa (Fig 4a) strongly suggestive of similar glycosylation patterns, and consistent with 3–4 N-linked sites. Both full-length sequences (Y529 and C529), in lysate format, bound C15 virus (Fig 4a), presumably through the same EC1-2 contacts. Surprisingly though, PNGaseF treatment did not affect this process. De-glycosylation, which certainly removed the N186 modification, did not prevent subsequent virus interactions.

Unlike transfected cells, transduced cells expressing CDHR3 Y529 (fCR3Y) show 2 forms of protein upon Western analyses (Fig 4b). The lower band (~100 kDa) was similar in size to that produced in transfected cells, or from C529 materials in any format (e.g. fCR3C transduced cells or C529 transfection). The upper band (~108 kDa) shifted when treated with neuraminidase indicating it displayed additional post-translational modification(s) involving supplemental sialylation. This shift was unique to CDHR3 Y529 when this protein was expressed constitutively in fCR3Y cells, instead of transiently (i.e. transfections). Sialic acids are frequently added or rearranged at the termini of N- and O- linked oligosaccharides, via α2–3 or α2–6 linkages [37,38]. The extra attachment(s) in Y529 was tested by the addition of biotinylated *Maackia amurensis* lectin II (MAL II) or biotinylated *Sambucus nigra* lectin (SNA), which respectively label proteins carrying α2–3 or α2–6 sialylated moieties. The Y529 upper band, when reacted with SNA-biotin was readily extracted with streptavidin beads, while very little protein was extracted with MAL II-biotin labeling (Fig 4c). Neither lectin extracted the CDHR3 Y529 lower band or any C529 material from the stably transduced cells. Therefore, the standard CDHR3 glycosylations (i.e. at N186, N384, N624) in transduced or transfected cells do not initially terminate in sialic acids, although one or more glycan branch termini, and perhaps as many as 20 (by estimated molecular weight), subsequently become sialylated by α2–6 linkages, when the Y529 sequence is transduced into cells. The extra sialic acid modifications in Y529 transduced cells are surface dependent, because when intact fCR3Y cells were labeled with biotin, the slower migrating, upper form of CDHR3 was observed only in the extracellular (biotin-labeled, bound) protein fraction and not in the intracellular (unlabeled, unbound) fraction (Fig 4d). Still, regardless of these modifications or how they originate, neither PNGaseF nor neuraminidase digestion of any expressed CDHR3 protein variants inhibited C15 binding (Fig 4b). The predicted glycan binding site on the C15a virion surface, apparently does not capture any N-linked or sialic acid glyco-moiety contributed by CDHR3.

### Virus binding to recombinant CDHR3 proteins

**Fig 5:**
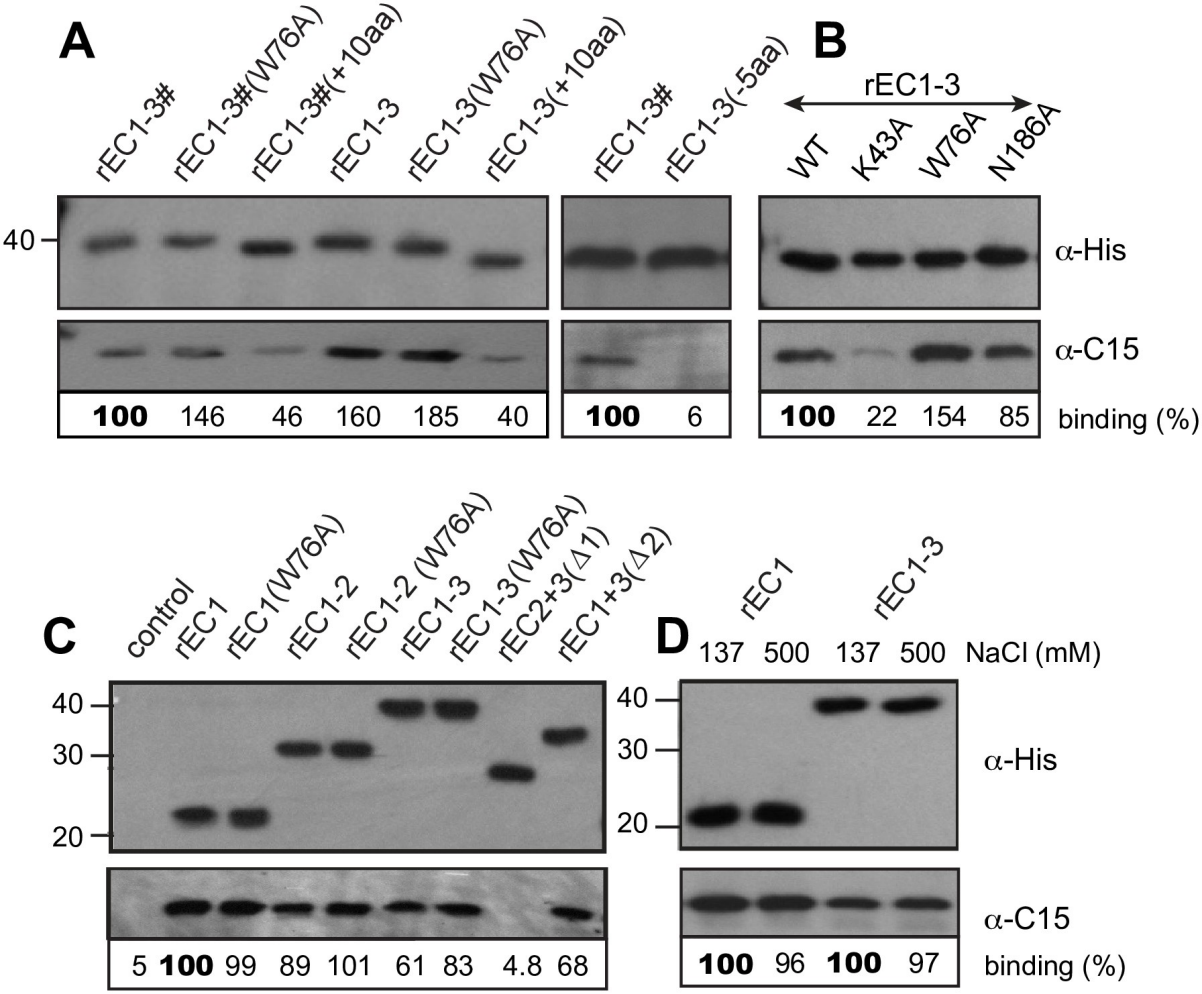
Recombinant CDHR3 proteins. Bacterially produced CDHR3 fragments (100 pmol) with the indicated C-terminal extensions (A), point mutations (B), or rEC deletions (C) were incubated with C15 virus (10^7^ PFUe). Immunoprecipitation with an α-His mAb was followed by Western analysis for bound proteins (α-His for CDHR3, and α-C15). As labeled here, “rEC1-3#” refers to a peptide with CDHR3 residues 20–345; “rEC1-3” is similar except these fragments also have a C345A substitution. (D) Similar to A, the α-His extracted complexes were treated with standard (137 mM) or high (500 mM) NaCl conditions before collection and Western analyses. Each panel (ABCD) is a separate experiment. Binding % is the observed C15 signal pixel count normalized to the recombinant protein (α-His) signal in the panel above (Total Lab 100) relative to positive control (100) in each unit.

The lysate binding assays mapped the segments and sequences of CDHR3 required for virus pull-down. In a formal sense though, those experiments could not preclude contributory partner interactions from other lysate components. Full-length CDHR3 proved insoluble when expressed in bacteria and not readily refolded. Accordingly, the next experiments focused on the EC1-3 domains. Like the successful lysate materials, the recombinant proteins were designed with amino-terminal FLAG-tags and carboxyl-terminal His-tags. The first constructions expressed native CDHR3 residues 20–345 (rEC1-3#). The amino-terminal signal sequence (residues 1–19) was not included because it prevented high-level protein expression in bacteria. Produced this way, >90% of the bacterial inclusion body material was the desired protein, which upon denaturation (urea) and refolding (see Methods) was capable of binding virus (Fig 5a). These first iterations though, had occasional solubility issues when residue C345, within rEC3-4 linker region, allowed spurious disulfide interactions. Shortening (-5aa) or lengthening (+10aa) the protein beyond this point was not nearly as effective, in terms of the tested virus binding efficiency, as a simple mutation of C345 to A345 within the plasmid backbone (e.g. rEC1-3). This context, after refolding and especially when augmented with an additional W76A mutation, was sufficient and highly effective in IP reactions (i.e. in buffer alone without other exogenous proteins), for purified C15 virus extractions. As with HeLa-produced proteins, W76A changes captured 50–60% more virus than the WT sequence (Fig 5b). K43A again proved inhibitory (22%), but surprisingly, in this recombinant format, where there was a denaturation and refolding protocol, N186A retained most of its reactivity with virus (85% of WT).

The next plasmid series assayed domain requirements, successively eliminating EC1, EC2 and EC3 (Fig 5c). The only inactive protein was that lacking EC1 [rEC2+3(Δ1)]. When the first domain was expressed alone or partnered with EC2 and/or with EC3, there was effective virus binding. The EC1 W76A mutation again seemed to improve virus binding whenever EC1 was linked to EC2 (rEC1-2) or to EC2 and EC3 together (rEC1-3), but not when EC1 was expressed by itself. Size exclusion experiments (S1 Fig) with Sephacryl S200 showed the rEC1 protein ran as single monomer whether or not W76 was mutated, suggesting that W76 may not behave like the characteristic tryptophans of classical cadherins and “strand-swap” to mediate EC1-dependent trans-dimerization. Interestingly though, all proteins containing an EC3 domain [rEC1-3, rEC2+3(Δ1), or rEC1+3(Δ2)] were completely excluded from the Sephacryl filtration and probably of multimeric order. The rEC1-2 WT and W76A proteins showed both monomers and higher order oligomers in these analyses. Since we consistently observed better virus-binding efficiency to rEC1 and rE1-2 (100 and 87%), which are monomers, relative to rEC1-3 or rEC1+3(Δ2) (61% or 68%), which form higher order oligomers, it is probable that the virus may prefer to bind monomers of CDHR3 when interacting with cells, too.

The virus captured in these assays was not by weak interactions, because for EC1 containing proteins, it resisted disruption with 500 mM salt (Fig 5d). Collectively, the results show that a direct interaction between purified virus and purified CDHR3 protein fragments does not require exogenous components, including glycosylation, and further suggest this binding is primarily mediated by the EC1 domain. N186 in EC2, and in fact, the whole EC2 domain itself, were not apparent contact requirements, but K43 (in EC1) undoubtedly plays a participating role.

**Fig 6:**
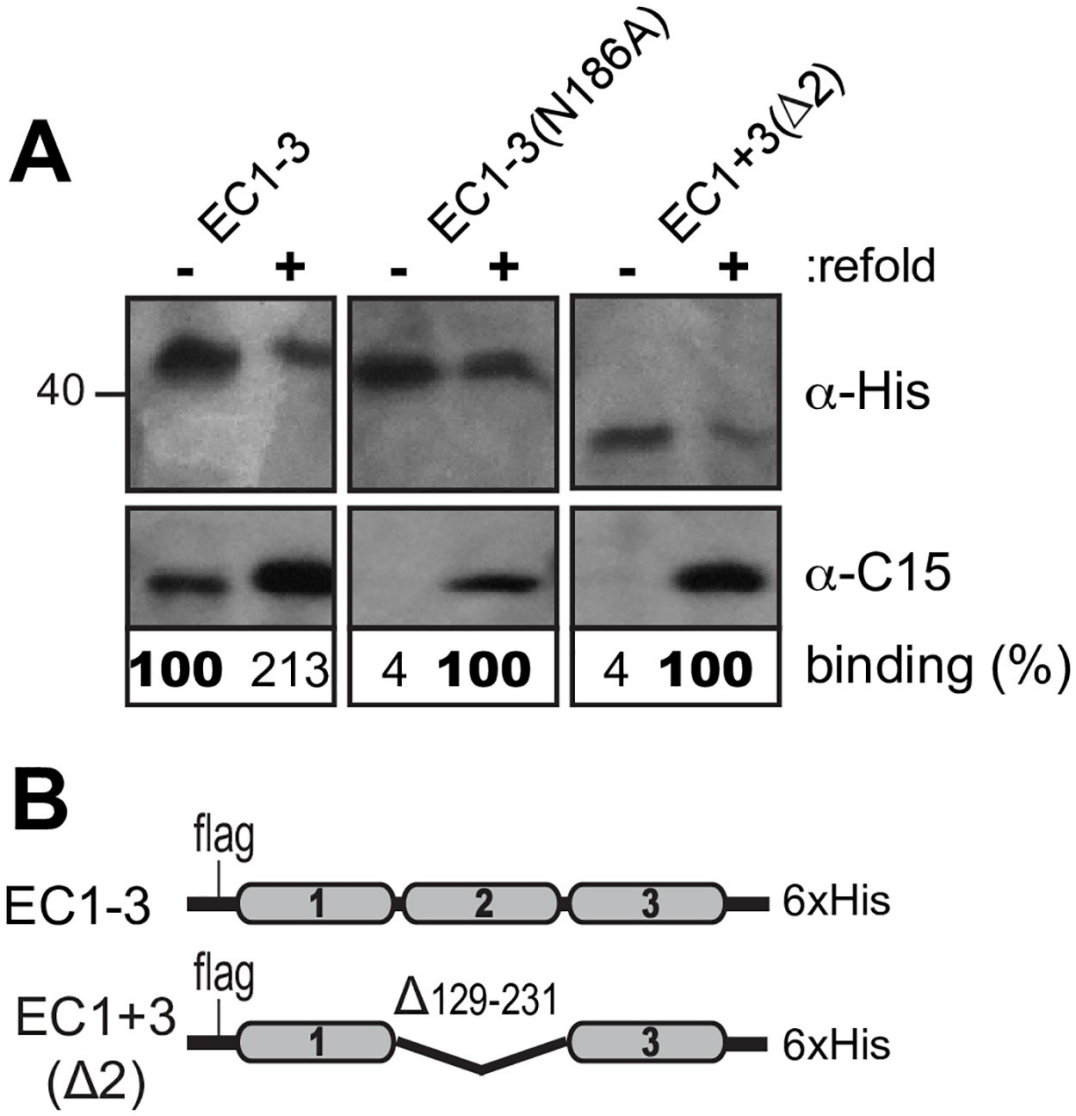
Misfolding of EC2. Lysates from HeLa cells transfected with cDNAs (B) as in Fig 1 were extracted with α-His mAb by IP to recover CDHR3 materials. (A) The recovered protein was refold (+) or not (-) according to the refolding protocol in Methods, then reacted with C15 virus. Immunoprecipitation and protein detection were with α-His mAb. Binding % for each protein pair is relative to the pixel count (Total Lab 100) of observed C15 signals normalized to the α-His protein levels (bold face).

To resolve why prokaryotic (bacteria) and eukaryotic (transfections) expressed proteins showed apparently different virus interaction requirements for the EC2 domain and for N186, transfection plasmids encoding EC1-3 (N186 or N186A), and EC1+3(Δ2), were introduced into HeLa cells (Fig 6b). The expressed proteins, extracted and recovered from cell lysates via their C-terminal His tags were reacted with C15 virus before and after a denaturation and refolding step, identical to that used for the bacterial materials. Refolding not only rejuvenated the EC1-3 protein (to 213%), it gave new activity to both other cell-expressed sequences. The ΔEC2 deletion, the N186A mutation, and presumably also R166A, as transfected lysates were inactive in these short contexts, and likely also in their full-length contexts (Fig 1) because they were somehow improperly configured. Allowed to refold, they then bound virus. The slight migration shift (~2 kDa) between EC1-3 with and without N186A is that expected if these proteins differed by a single glycosylation unit. Therefore, in HeLa cells, the N186 glycan linkage may be required for proper domain folding.

### A requirement for calcium

**Fig 7:**
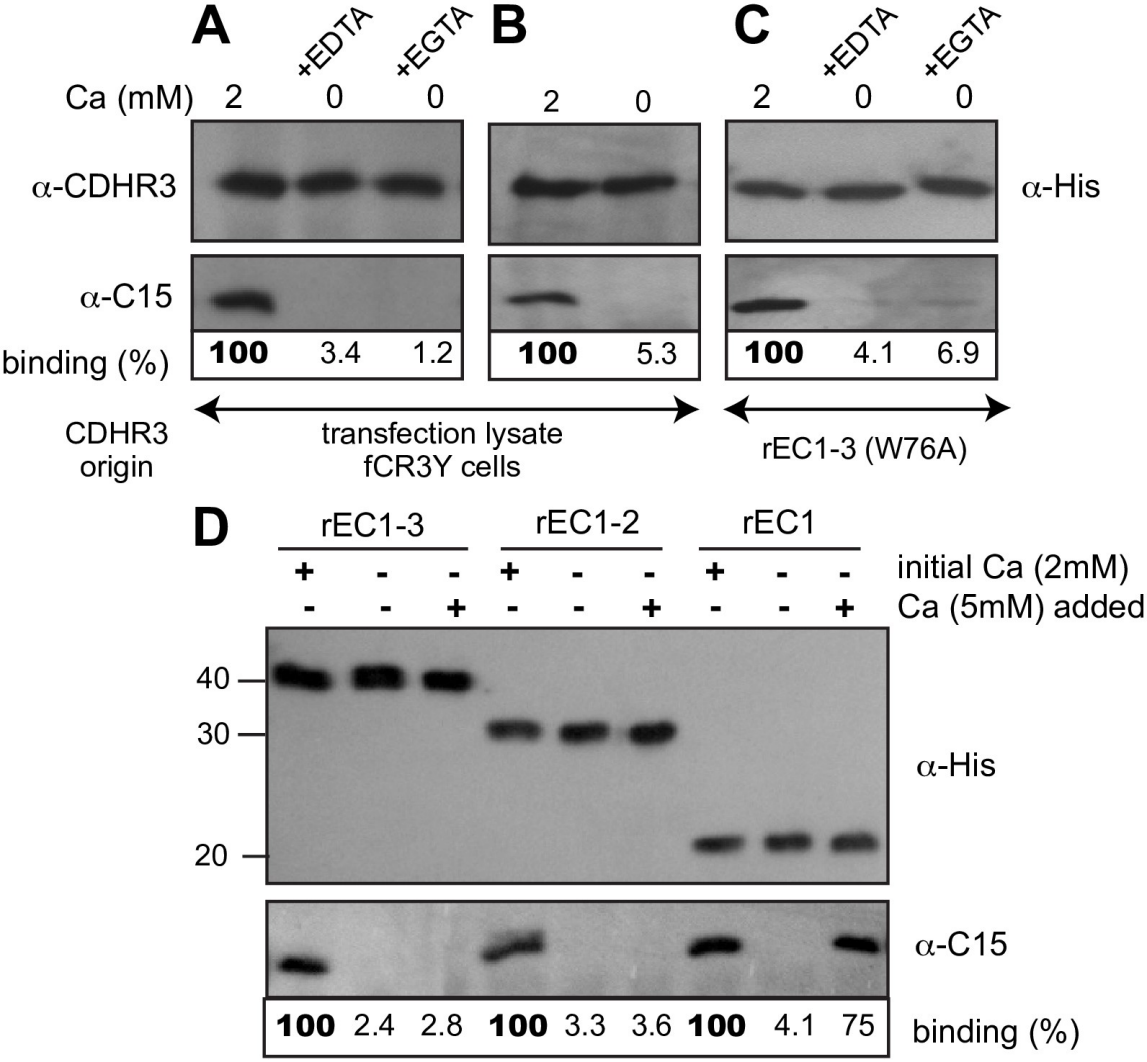
Dependence of CDHR3 and C15 binding on calcium. Lysates transfected with cDNAs encoding Y529 CDHR3 protein were reacted with C15 virus in buffer with 2mM calcium, 2mM EDTA or EGTA (A), or in calcium-free buffer (B) and immunoprecipitated with an α-CDHR3 mAb reactive with the cytoplasmic domain. (C) Purified rEC1-3 protein (100 pmol) was reacted with C15 virus in buffer with 2mM CaCl_2_ or 2 mM EDTA or EGTA. (D) rEC1-3, rEC1-2, and rEC1 (100 pmol) protein were first reacted with C15 virus in buffer with or without 2 mM CaCl_2_ for 1 h at 25 and then incubated another 2 hrs following the addition of 5 mM CaCl_2_ to designated samples. Immunoprecipitation was with an α-His mAb. Each panel (ABCD) is a separate experiment. Binding % is the observed C15 signal pixel count normalized to the CDHR3 protein (α-His) signal in the panel above (Total Lab 100) relative to positive control (100) in each unit.

The rod-like arrangement of EC domains in classical cadherins is dependent upon calcium binding to multiple acidic clusters at the inter-domain junctions. Removal of calcium causes the proteins to collapse into more condensed structures [31,39,40] and can also affect the oligomerization status when the domains no longer orient properly [41,42]. The CDHR3 structure model predicts analogous acidic clusters at each EC junction [23], but modeling cannot accurately anticipate the exact ion placement or count. In the lysate assay with full-length HeLa-produced CDHR3, the addition of EDTA or EGTA reduced the virus binding to background levels (Fig 7a). Remarkably, the same effect was achieved by simply diluting the lysate into a buffer lacking calcium (Fig 7b). This was also observed with recombinant proteins, in that EDTA, EGTA (Fig 7c) or simple omission of calcium (Fig 7d, “0 initial”) prevented virus extraction by IP. This suggests that one or more of the required ions must be able to diffuse from the EC junction(s), leading to a conformational change in the protein, or its oligomeric state, as required by the virus. When E-cadherin, is depleted of calcium, the protein collapse is reversible [39]. When calcium was added back (for 2 hrs) to soluble rEC1-3 or rEC1-2 that had been diluted into a calcium-free buffer, these proteins still could not bind C15 (Fig 7d). However, virus binding to rEC1 could be restored upon re-addition of calcium. Likely, the diffusible, stabilizing calcium(s), normally at the interface of EC1 and EC2 can be rebound if EC1 is a monomer, and not linked to EC2. The calcium site must become sterically impaired by depletion-induced conformational changes at this junction when EC1 is linked to EC2 (or EC3).

### Recombinant CDHR3 inhibition of RV-C infection

**Fig 8:**
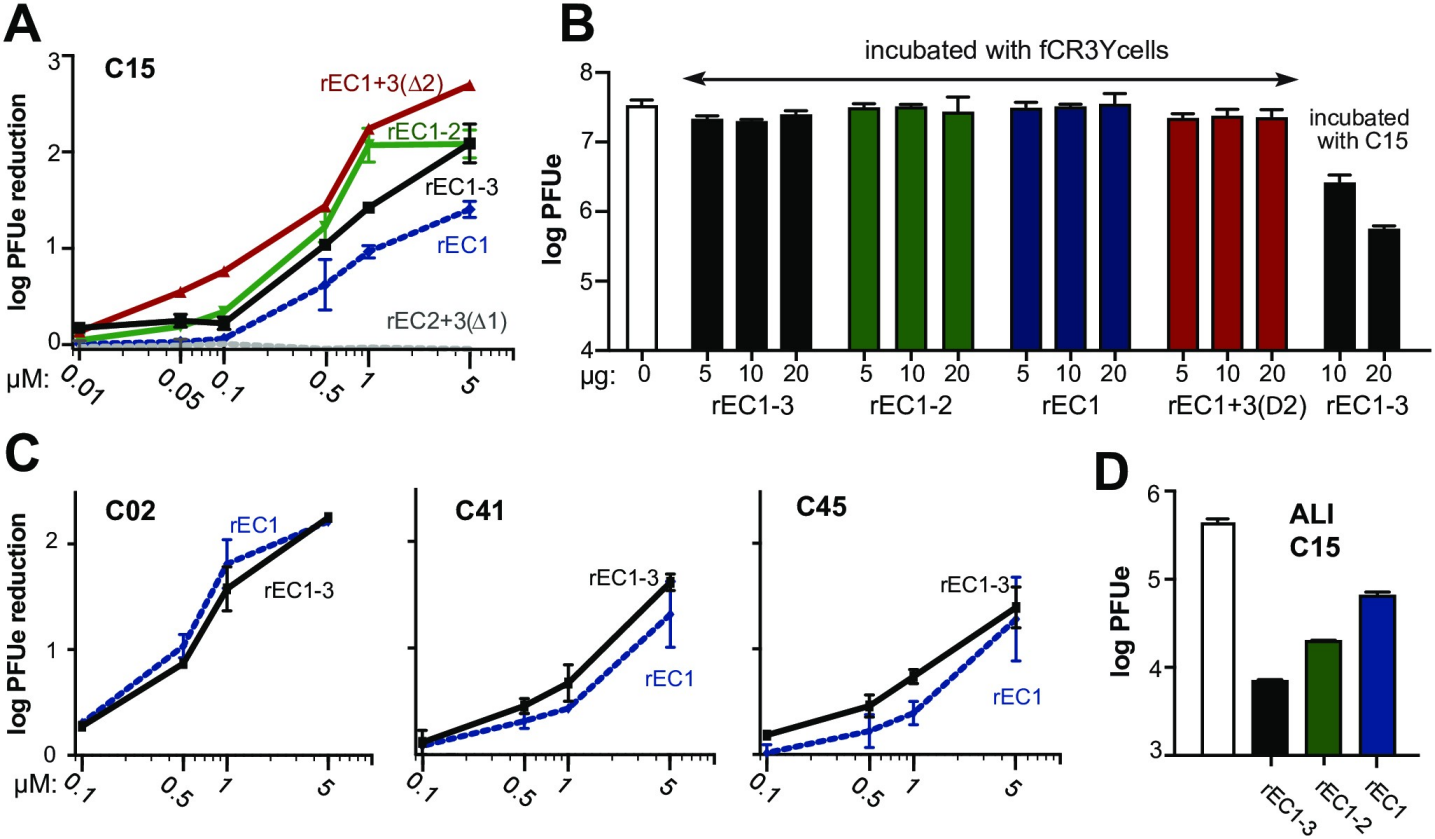
Inhibition of RV-C infection. (A) C15 virus was preincubated with increasing amounts of the specified recombinant protein (0.05–5 μM) before infection of fCR3Y cells. Cells were washed after attachment and samples collected 24 hpi (triplicate). Virus titers (PFUe) were measured by qPCR. (B) fCR3Y cells were preincubated with or without the specified recombinant protein (5–20 μg) before being washed and infected with C15 virus, or with C15 virus pretreated with rEC1-3 (right lanes). Samples (duplicate) were collected 24 hpi and viral loads measure by qPCR. (C) fCR3Y cells were infected with C02, C41, or C45 virus (10^7^ PFUe) that had been pretreated with the indicated recombinant protein as in A. Error bars are average of duplicate samples collected 24 hpi. (D) C15 (10^6^ PFUe) was preincubated (1h) with or without 1 μM of the specified recombinant protein (in 50 μL) before infection of differentiated nasal epithelial ALI cultures. Single replicate samples were collected 24 hpi for measurement of viral loads (qPCR).

Soluble ICAM-1 and LDLR materials can bind their appropriate RV-A and RV-B isolates to inhibit virus infection of susceptible cells [43–45]. The soluble recombinant CDHR3 protein panel was tested for its ability to inhibit C15 infection of stably transduced fCR3Y cells. When pre-incubated with virus before inoculation of cell monolayers, all recombinant proteins containing EC1 inhibited infection in a dose dependent manner (Fig 8a). Native cadherin proteins commonly form dimers or higher order oligomers on the surfaces of cells [18,46]. Although small amounts of the active proteins were inhibitory, the observed recombinant CDHR3 effects were highest when up to 5 μM of protein were added to each sample (10^6^ plaque forming unit equivalent (PFUe) of C15). Thus, the mode of observed inhibition could have been due to recombinant protein blocking of CDHR3 receptor sites on each particle, or to direct association of these proteins with the native CDHR3, perhaps through oligomerization, masking the good receptors on the cell surface. The possibilities were distinguished by pre-incubation directly with cells, or directly with virus, before infectivity was tested. Pretreatment of fCR3Y cells with any recombinant protein at any of 3 concentrations, did not protect them from subsequent C15 infection (Fig 8b). Inhibition required the virus to be pretreated with soluble CDHR3, and that protein needed to encode EC1. The same was true when the tests used additional strains of RV-C (Fig 8c). Just like C15, C02, C41, and C45 infections were inhibited by soluble CDHR3 rEC1 and rEC1-3, in a dose dependent manner. Finally, we tested the ability of soluble CDHR3 to protect airway epithelial cells, which naturally express CDHR3 and are the primary site of RV infection, by using differentiated nasal epithelial ALI cultures. In a preliminary single-replicate experiment, C15 infection of these cells was also reduced by 1 μM rEC1, rEC1-2, and rEC1-3 (Fig 8d).

## Discussion

The binding of a virus to accessible external receptor(s) is an initiating step in host cell entry. CDHR3 cell surface display, mediated by the Y529 variant SNP of this gene, is required for optimal RV-C entry into cells [15]. The dominant human allele, encoding C529, shows much lower protein surface expression in transfected cells and consequently poorer cell-binding interactions with virus. For homozygous or heterozygous Y529 human carriers, especially children, there is a correlate higher rate of virus-induced asthma exacerbations [25]. The current study examined three important questions concerning these observations. First, we asked if there might be measurable discrepancies between the Y529 and C529 proteins, in addition to surface display, that could influence virus interactions? Second, if RV-C did bind directly with either or both proteins, could we devise reproducible assays to map the elements of CDHR3 or its glycosylation format that might be required for this interaction? Third, assuming CDHR3 like the ICAM-1 and LDLR receptors of the RV-A and RV-B could be isolated in a cell-free format, would such materials independently react with virus and potentially inhibit infections?

Cadherin proteins share a common architecture in that the tandem repeat EC domains (EC1-6 for CDHR3) assume a rigid, slightly curved elongated structure, anchored like a waving stalk in the cell membrane. The C-proximal cytoplasmic domain does the anchoring. The N-proximal distal domains (e.g. EC1-3) usually confer adhesion properties by mediating dimer formation or higher order arrangements [26]. The linked EC orientations and even the folding of individual EC units depend on multiple calcium ions bound at various K_d_, between the EC junctions. The first challenge in examining CDHR3 was to devise a virus-binding assay that was not cell surface dependent. In transfected or stably transduced cells, the intracellular protein pools are frequently much larger than that which is membrane anchored [15]. Clarified cell lysates proved a ready source of assay materials, and we found no CDHR3 sequences, fragments or conditions that required cell anchoring for demonstrable reactivity with virus. Tested this way, C529, Y529 and H529 proteins were equivalently capable of virus IP (Fig 2a). The H529 sequence was tested as a curiosity because it is one of the only (non-human) variations of the highly conserved ancestral Y529 allele [24].

The basic N-linked glycosylation sites of human C529 and Y529, mapped to N186 (EC2), N384 (EC4), and N624 (EC6). These proteins migrated equivalently on gels by molecular weight, indicating both must undergo similar Golgi transport and modification pathways on the way to the cell surface. But once there, the Y529 abides, and can be labeled with biotin, while the C529 somehow withdraws, or undergoes a faster surface cycling pattern and is not labeled with biotin. In mature, plated stably transduced cells (fCR3Y), the presumed longer surface “hang time” of constitutively expressed protein apparently then permits Y529 to undergo additional multiple sialyations with α2–6 linkages. Transfected cells, even for Y529 do not have detectable amounts of these modifications, perhaps because the signal strength is masked by the much larger cytoplasmic pool created by overexpression, or because Y529 does not have time to fully surface-mature within 24 hrs post-transfection.

Surprisingly, none of these parameters proved relevant to virus binding. Whether the materials were from transfections, transductions, or bacterially produced, virus could be extracted with almost any CDHR3 format, including after de-glycosylation, as long as a properly reconstituted EC1 domain was present. In fact, EC1 alone was sufficient to bind virus (Fig 5c). Initially, a caveat to this was, that when expressed after transfection, ΔEC2, as well as EC2 point mutation derivatives N186A and N186Q also did not react with virus. Yet in a recombinant bacterially-derived format, rEC1+3(Δ2) was entirely active. This dichotomy, quite clearly had its origin in cell-dependent protein folding. During cell expression and Golgi maturation, EC2 and its glycosylation status at N186 must help the native EC1-2 segments fold. The bacterially-produced recombinant proteins (Fig 5), or similar sequences synthesized in HeLa cells, all presented a virus-acceptable EC1 if they underwent an artificial refolding step through denaturation and dialysis (Fig 6).

The R166A mutation, also in EC2, models immediately adjacent to the EC1-EC2 junction, a region predicted to have 11 Asp and Glu calcium-chelating residues within 5–9 Å of its location (Fig 2b). Virus binding proved exquisitely sensitive to the presence of calcium, whether the CDHR3 materials were derived from cells or bacteria (Fig 7). Not only could EDTA or EGTA prevent interactions, a simple dilution into calcium-free buffer had an equivalent effect. This property is characteristic of cadherins. E-cadherin binds 3 calcium ions between the EC1 and EC2 domains, two at 330 μM K_d_ and a third at a much higher 2 mM K_d_, a value close to the extracellular calcium concentration [41,47]. For E-cadherin, calcium depletion and consequent protein unfolding is reversible [39], but this does not appear to be true for CDHR3 because rEC1-3 and rEC1-2 could not adopt a virus-acceptable conformation when calcium was added back after dilution into a calcium-free buffer (Fig 7d). Interestingly, the readdition of calcium did facilitate proper protein refolding of the singular rEC1 protein. Presumably, CDHR3 needs calcium to fold properly, maintain that fold, and at least 1 or more crucial ion(s) at the EC1-2 junction are irreversibly lost by simple diffusion, likely due to conformational changes in EC2 that sterically prohibit proper calcium re-coordination. This point has also been proposed as functionally germane to the C529/Y529 surface display phenotypes [23]. Similar to R166 at the EC1-2 junction, Y529 is part of the EC5-6 junction. If conversion to C529 were to influence the inter-domain calcium binding and the consequent topography of stalk, an improperly configured protein would be withdrawn from the cell surface, as are similar calcium-depleted cadherins in their natural cycle [48,49]. We are currently testing this hypothesis by determining the NMR structures of cadmium-labeled CDHR3 rEC4-6 domains.

An innate property of all cadherins on cell surfaces or in solution is to find dimeric and/or oligomeric partners. Most certainly the EC3 domain of our proteins promotes self-association because, although soluble upon refolding, proteins containing this domain flow directly through a Sephacryl S200 column and therefore are of dimer or higher order (S1 Fig). There is only one native, internal CDHR3 cystine disulfide, linking C566 to C592 within EC6 [23], but the rEC1-3 proteins formed spurious, exogenous disulfides upon refolding, unless C345 was mutated. Even with this change, the protein panel of rEC fragments was still capable of self-assembly. We are currently working with several physical sorting and crystallographic techniques to define the exact nature, order and residue contributors of these self-interactions. Consistent with what is known for other cadherins, we presumed the W76A mutation acts positively by reducing *(trans*) oligomer states, thereby freeing more EC1 units for productive virus binding. However, the rEC1 protein appears to be monomeric with or without this mutation. While W76 may not mediate EC1-EC1 dimerization like the tryptophans of other cadherins, it could still be involved in inter-protein interactions with other CDHR3 EC domains. Alternatively, W76 may lie within or near the virus-contact interface, and elimination of the bulky aromatic side chain may allow tighter virus binding. Ongoing (NMR and cryoEM) structural studies with rCDHR3 proteins should provide some insight on the positive effects we observe for the W76A mutant.

When the panel of recombinant proteins was tested in infectivity inhibition assays, all inhibited virus replication in fCR3Y cells in a dose-dependent manner except rEC2+3(Δ1) (Fig 8a), which also could not IP virus. We have initiated a collaborative cryoEM determination of the C15 virus complexed with rEC1-3 (with W76A) and rEC1. It will take time for those data collections to achieve sufficient resolution, although we expect they will confirm the protein interactions and format predictions described here. Our original computational docking model suggested elements of both EC1 and EC2, including an N186 glycan, made virus contact [28]. We now know only the EC1 part of this prediction is true. It is more likely that dimers of CDHR3, probably mediated by EC3 *cis* interactions, present dual EC1 domains to the virus. The unique EC1 contacts, perhaps including K43, are the primary determinants. EC2 is not required, nor is its glycosylation, except perhaps to help the protein fold properly. Apparently, soluble recombinant proteins contributed in this format are sufficient to inhibit infection of stably transduced fCR3Y HeLa and differentiated nasal epithelial ALI cultures. The observed inhibition phenomenon was common to 4 tested genotypes of virus, indicating that all RV-C virions likely share the same receptor landing pad and would respond to these soluble recombinant CDHR3 sequences in a similar manner. We believe these new reagents have great potential utility in RV-C research as useful tools for investigating CDHR3 biology and function, possibly with future antiviral applications.

## Materials and methods

### Eukaryotic expression plasmids and transfections

Plasmids for transient eukaryotic expression of FLAG-tag-CDHR3 C529 and Y529 variants (pCDHR3-FLAG-C529 and pCDHR3-FLAG-Y529), which have a FLAG-tag (DYKDDDDK) inserted between the CDHR3 signal sequence (residues 1–19) and its EC1 (residue 20), were previously described [15]. The protein numbering system is from GenBank: AIC58018. Additional plasmids encoding single point mutations (K43A, W76A, F152A, R166A, R182A, N186A, N186Q, and Y529H) were engineered by two-step PCR into the pCDHR3-FLAG-Y529 plasmid. Analogous units for the expression of various FLAG-tag CDHR3 EC deletion mutants, which included additional carboxy-terminal 6x His tags, were engineered within pHLsec vectors (generously provided by Yue Liu, Michael Rossmann) based on pLEXm plasmid backbones [50]. Pilot experiments indicated that when this vector’s innate secretory sequence was linked to the native CDHR3 signal sequence, protein expression was inhibited in transfected cells. Therefore, the 5’ regions of the CDHR3 EC deletion sequences, encoded only the native signal sequence and its inserted FLAG-tag, as engineered into pHLsec vector backbones between BamHI and KpnI restriction sites. pHL-FLAG-CDHR3-His constructs included: WT (CDHR3 residues 1–885, Y529), ΔEC1 (1–885, Δ27–124), ΔEC2 (1–885, Δ129–231), ΔEC3 (1–885, Δ230–334), EC1-6 (1–689), EC1-3 (1–345), EC1-2 (1–237). Transfection protocols used 3 μg cDNA reacted with lipofectamine 2000 (3 μL, Invitrogen) in Opti-MEM media (Invitrogen) according to manufacturer’s recommendations, and plated HeLa cells (ATCC CRL-1958 in Eagle’s medium, 10% NBCS; 6-well dishes; 37°C) grown to 80% confluence. The cells were incubated 24 h (37°C under 5% CO_2_) before collection, lysis and immunoprecipitation assays.

### Transformed cell lines

Amplicons containing the C529 and Y529 FLAG-tagged variants of CDHR3 were amplified by PCR from pCDHR3-FLAG cDNAs and then ligated into MIGR1-based IRES-neo retroviral plasmids (NG) which express neomycin resistance [51,52]. Viral vector generation required 293T cell transfection with pNG-FLAG-CDHR3 plasmids (4 μg), pMDGag-Pol (4 μg, packaging plasmid), and a vesicular stomatitis virus G protein-encoding (VSV-G) envelope plasmid (2 μg) in 500 μL of Opti-MEM with 20 μL of polyethylenimine. The transfection medium was replaced 12 h post-transfection, then subsequently harvested and filtered (0.45 μm, at 48 h). After infection of HeLa cells (ATCC CRL-1958) with this material by spinoculation [53] and incubation for genome integration, stably transformed cells were selected with G418 (400 μg/mL, Geneticin) and cloned. The cells were maintained in suspension culture [37°C; Eagle’s medium, 10% newborn calf serum (NBCS), 2% fetal bovine serum (FBS) under 5% CO_2_]. The final transduced HeLa cell lines expressing full-length C529 and Y529 FLAG-tagged variants of CDHR3 were designated fCR3C and fCR3Y, respectively.

### CDHR3 N-linked glycosylation

Cells (2x10^6^) transfected with pCDHR3-FLAG-Y529 were lysed in PBS (100 μL, 0.5% SDS and 40 mM DTT) and then heated (10 min, 95°C). The denatured lysate was diluted 2-fold into PBS and 1% NP40 and then equivalent samples were incubated with or without PNGaseF (1U, 1 h, 37°C, Sigma F8435). The proteins (25 μL lysates) were fractionated by SDS-PAGE and visualized by Coomassie Brilliant Blue staining. Bands corresponding to glycosylated (~100 kDa) and de-glycosylated (~93 kDa) CDHR3 gel regions were cut out and submitted for analysis to the Mass Spectrometry/Proteomics facility at the University of Wisconsin Biotechnology Center for MS/MS analysis after in-gel trypsin digestion.

### Recombinant CDHR3

Bacterial plasmids for the expression of various CDHR3 rEC domains, linked to amino-terminal FLAG-tags and carboxy-terminal 6x His tags, were constructed. The rEC1 segment encoded residues 20–130, rEC1-2 encoded residues 20–237, rEC1-3 encoded residues 20–345, rEC2+3(Δ1) encoded residues 20–345 (Δ27–124), rEC1+3(Δ2) encoded residues 20–345 (Δ129–231), rEC1-3(+10aa) encoded residues 20–355, and rEC1-3(-5aa) encoded residues 20–340. These units were amplified by PCR from the pHL-FLAG-CDHR3-His cDNAs described above and then ligated into pET11a vectors between the NheI and BamHI restriction sites. To prevent spurious disulfide formation, most plasmids encoding EC3 segments had a point mutation converting Cys345 to Ala345 (C345A). Additional point mutations were engineered by standard, primer-directed two-step PCR. Escherichia coli BL21(DE3) LysS cells, transformed with each plasmid were induced with IPTG (isopropyl-β-d-thiogalactopyranoside) for recombinant protein expression. The cells were collected by centrifugation, resuspended in lysis buffer (20 mM Tris, pH 8.0, 137 mM NaCl, 1% Triton-X100), and sonicated. The majority of recombinant material was insoluble and collected by centrifugation (20,000 × *g* for 45 min at 4°C). The pellets were washed (1 M NaCl, then 1 M urea, then water) and solubilized (6 M urea, 20 mM Tris, pH 8.0, 137 mM NaCl for 1 h at 25°C or O/N at 4°C). After clarification (20,000 × *g* for 45 min at 4°C), the supernatant of rEC1 and rEC1-2 proteins were purified under denaturing conditions (6 M urea, 20 mM Tris, pH 8.0, 137 mM NaCl) on HisTrap FF columns (GE Healthcare). After elution with 200 mM Imidazole, the proteins were diluted (to 0.1–0.2 mg/mL) in the solubilization buffer (above) supplemented with 3 mM CaCl_2_ and then dialyzed (4 times, 8–12 hrs each, against 20 mM Tris, pH 8.0, 137 mM NaCl, 3 mM CaCl_2_, 2 mM β-mercaptoethanol). Proteins containing the EC3 domain [rEC1-3, rEC2+3(Δ1), and rEC1+3(Δ2)] bound poorly to the HisTrap FF columns even under denaturing conditions so the clarified supernantants were diluted and refolded as described above. Refolded proteins were concentrated using Amicon Ultra centrifugal filters.

### Viruses

Recombinant RV-C isolates C02, C15, C41, and C45 were produced by transfecting full-length T7 RNA transcripts synthesized *in vitro* (Ribomax, Promega) from linearized plasmid cDNAs into HeLa cells [54]. The C02 and C45 sequence encoded a D41K substitution in protein 3A, to increase virus replication in these cells. In contrast to the more prolific, HeLa-adapted C15a sequence, these recombinants do not encode a T125K substitution in capsid protein VP1. Therefore, like their parental clinical isolates, they do not bind heparan sulfate and their cell interactions are entirely dependent upon CDHR3 presentation [54]. Virus purification was by centrifugation of cell lysates through 30% sucrose cushions as described [55].

### Virus binding/Immunoprecipitation assays

CDHR3 proteins expressed in transfected or stably transformed HeLa cells (~ 2 x10^6^ cells scraped, collected in PBS, pelleted) were harvested 24 h after transfection or plating. The cells were pelleted (1.5 min at 1500 × *g*), resuspended and then lysed (350 μL, 20 mM Tris, 137 mM NaCl, 2 mM CaCl_2_, 2 mM PMSF, 1% Triton x-100). The lysates were clarified (16,000 × *g*, 20 min) and then incubated with sucrose purified C15 virus (10^7^ PFUe) and with antibody (0.8 μL, α-CDHR3, HPA011218, Sigma; or 1 μg α-His Tag, HIS.H8, Millipore) overnight at 4°C before being reacted with protein-G sepharose beads (1 h, 25°C). When required, glycosylases PNGaseF (1U, Sigma F8435) or neuraminidase (0.04U, Sigma 10269611001) were included during the overnight incubations. After reaction and collection, the beads were washed (3x, lysis buffer) and bound proteins eluted with SDS (boiling), before SDS-PAGE fractionation and visualization by Western blot analysis. For experiments with bacterially-expressed materials, the refolded protein samples (100 pmol) were incubated with virus (10^7^ PFUe, 1 h, 25°C) and with the α-His Tag antibody (350 μL, 20 mM Tris, 137 mM NaCl, 2 mM CaCl_2_, 1% Triton x-100) before reactions with protein-G sepharose beads and treatment as above.

### Biotinylation assays

The sialylation status of CDHR3 expressed in fCR3Y or fCR3C was tested by incubating (1h, 25°C) cell lysates (~ 2 x10^6^ cells in 300 μL, PBS 1% TritonX-100) with 5 μg biotinylated *Sambucus nigra* lectin (SNA) or *Maackia amurensis* lectin II (MAL II, Vector Labs) before addition to streptavidin beads (1 h, 25°C). Collected beads were washed (3x PBS) before the bound protein was eluted (in 30 μL 2% SDS, with boiling), fractionated by SDS/PAGE and then visualized by Western blot analysis. Extracellular expression of CDHR3 was examined by treating plated cells (~ 2 x10^6^ per sample) with EZ-Link Sulfo-NHS-Biotin (2 mM, ThermoFisher, in PBS for 1 h at 25°C). The cells were then washed (3x, 50 mM Tris, pH 8.0; 3x PBS), harvested, lysed (300 μL, PBS 1% TritonX-100). The clarified lysates were reacted with streptavidin beads (1h, 25°C). The bound samples were processed for protein detection as above.

### Western analyses

After SDS-PAGE resolution, proteins were electro-transferred to polyvinylidene difluoride membranes (Immobilon-P, Millipore). The membranes were blocked (1 h, 10% NFD milk in TBST: 20 mM Tris pH 7.6, 150 mM NaCl, 0.5% Tween20) then incubated with a primary antibody (1% NFD milk in TBST, overnight, 4°C) before washing (3x TBST) and reaction with an appropriate secondary antibody (1 h, 20°C). Commercial antibodies included: α-CDHR3 (rabbit Ab HPA011218 IgG, Sigma, 1:2000), α-FLAG (rabbit mAb F2555, IgG, Sigma, 1:2000), α-His Tag, (murine mAb HIS.H8 IgG, Millipore, 1:4000), HRP-conjugated α-mouse IgG (goat Ab A1068, Sigma (1:4000), and HRP-conjugated α-rabbit IgG (goat Ab A0545, Sigma, 1:4000). α-C15 (18C4 and 30C12, 1:5000) are custom murine mAbs (1 mg/mL,GeneScript) raised to the VP1 “finger” peptide sequence [28] characteristically exposed on the surface of this RV-C virion structure. For band visualization, the membranes were rinsed (3x, TBST), incubated (1 min) with enhanced chemiluminescence substrate (GE healthcare) and then exposed to film.

### Infection inhibition assays

Typically, virus (3x10^6^ PFUe) was incubated (1 h, 25°C) with or without refolded recombinant CDHR3 protein (0.01 to 5 μM) in binding buffer (100 μL, 20 mM Tris pH 8.0, 137 mM NaCl, 2 mM CaCl_2_) before dilution into Eagle’s medium (250 μL). Inoculation was onto plated, stably transformed fCR3Y cells. After attachment (30 min at 25°C, 15 min at 34°C), the cells were washed (2x with PBS) to remove unattached virus and incubated (24 h at 34°C) before harvest (lysis in 350 RLT buffer, Qiagen) and assessment of virus replication. Alternatively, the cells were directly incubated with recombinant CDHR3 protein (0–20 μg in 100 μL binding buffer, diluted into 250 μL Eagle’s medium, 30 min 25°C, then 15 min 34°C) and then washed (2x, PBS) before being exposed to virus as above. The cells were washed (2x with PBS) to remove unattached virus, before incubation (24 h at 34°C), harvest and virus measurements. Viral loads (PFUe) were determined by RT-qPCR according to standardized RNA preparations after total RNA extraction from harvested cells (RNeasy Mini kits, Qiagen). The RT-qPCR reactions used Power SYBR Green PCR mix (Life Technologies) and RV-C specific primers as previously described [10]. For experiments with differentiated primary nasal epithelial cells, cells were obtained from nasal turbinates using ASI Rhino-Pro curette (Arlington Scientific) and cultured at air-liquid interface in collagen-coated Transwell polycarbonante inserts as previously described [10,11]. Fully differentiated cultures (2 months old) were washed with PBS and inoculated with C15 virus (10^6^ PFUe) preincubated (1 h, 25°C) with or without 1 μM recombinant CDHR3 protein (50 μL 20 mM Tris pH 8.0, 137 mM NaCl, 2 mM CaCl_2_). After attachment (30 min at 25°C, 15 min at 34°C), the cells were washed (3x with PBS) to remove unattached input virus and incubated (24 h at 34°C) before harvest for assessment of virus replication as described above.

## Supporting information

S1 Fig Gel filtration chromatography of rEC CDHR3 proteins. (A) Chromatogram of Gel-Filtration standards: Blue Dextran 2000 (1 mg, D4772, Sigma), BSA (0.5 mg, 23209, Thermo Scientific), Ovalbumin (0.8 mg, A7642, Sigma), Carbonic Anhydrase (1 mg, C2273, Sigma), Cytochrome C (1 mg, C7150, Sigma) separated on HiPrep 16/60 Sephacryl S200 column (GE Healthcare). (B) Chromatograms of the specified refolded rEC CDHR3 protein preparations (0.5–1.5 mg) run on HiPrep 16/60 Sephacryl S200 column. All standards and samples were run at 1 mL/min in 20 mM Tris (pH 8.0), 137 mM NaCl, and 2 mM CaCl_2_.
(TIF)
