# Supplementary figures and images for: CDHR3 extracellular domains EC1-3 mediate rhinovirus C interaction with cells and as recombinant derivatives, are inhibitory to virus infection
Source: PLoS Pathog. 2018 Dec 10;14(12):e1007477. doi: 10.1371/journal.ppat.1007477 (PMC6301718; doi:10.1371/journal.ppat.1007477)

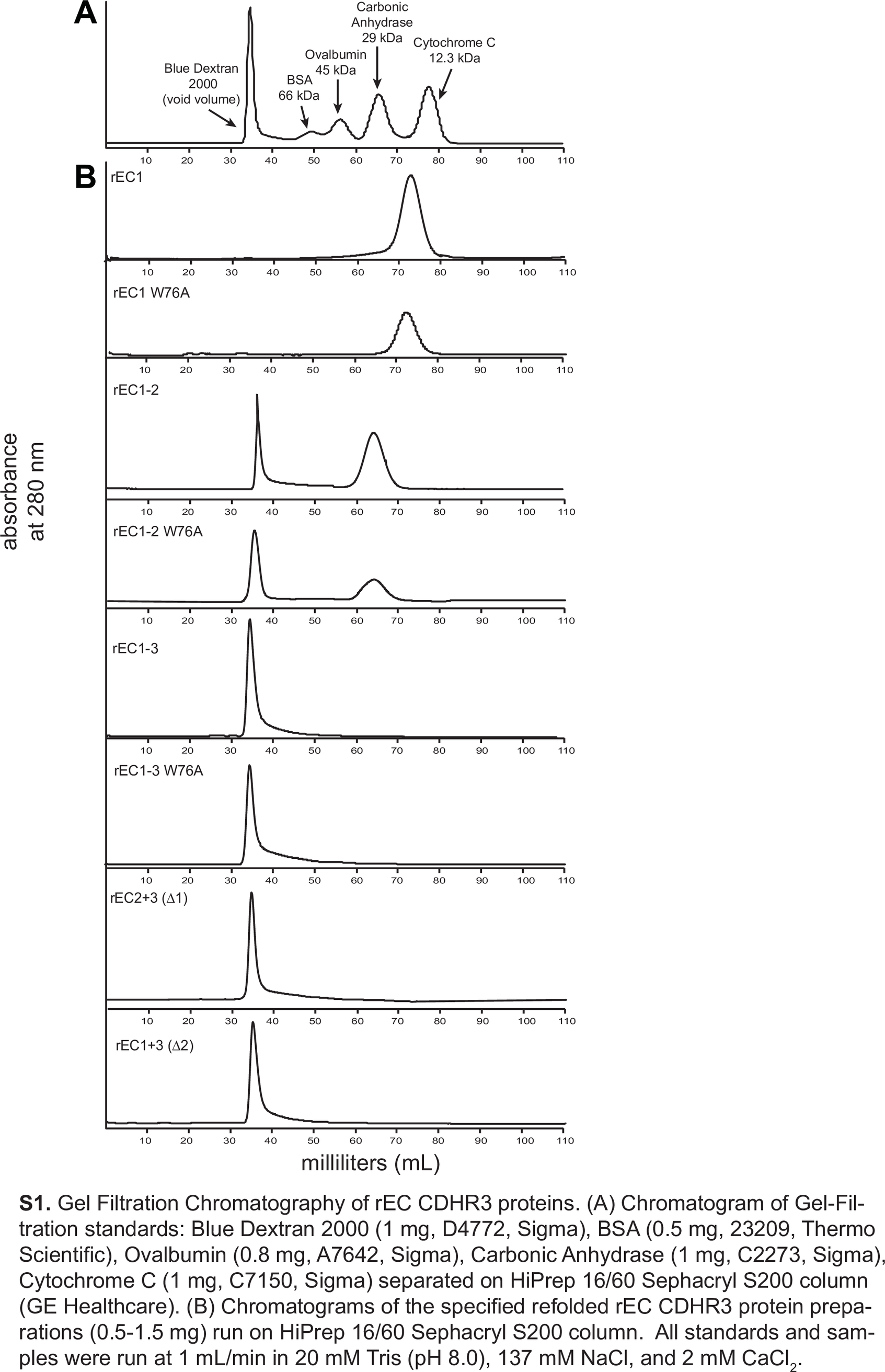

Supplement: S1 Fig — (A) Chromatogram of Gel-Filtration standards: Blue Dextran 2000 (1 mg, D4772, Sigma), BSA (0.5 mg, 23209, Thermo Scientific), Ovalbumin (0.8 mg, A7642, Sigma), Carbonic Anhydrase (1 mg, C2273, Sigma), Cytochrome C (1 mg, C7150, Sigma) separated on HiPrep 16/60 Sephacryl S200 column (GE Healthcare). (B) Chromatograms of the specified refolded rEC CDHR3 protein preparations (0.5–1.5 mg) run on HiPrep 16/60 Sephacryl S200 column. All standards and samples were run at 1 mL/min in 20 mM Tris (pH 8.0), 137 mM NaCl, and 2 mM CaCl2. (TIF) [file ppat.1007477.s001.tif]
